# Ground beetles of the Ukraine (Coleoptera, Carabidae)

**DOI:** 10.3897/zookeys.100.1545

**Published:** 2011-05-20

**Authors:** Alexander Putchkov

**Affiliations:** Institute of Zoology NASU, ul. B. Khmelnitzkogo, 15, Kiev-30, MSP, 01601, Ukraine

**Keywords:** Coleoptera, Carabidae, distribution, geographic region, Ukraine

## Abstract

A review of the ground beetles of the Ukrainian fauna is given. Almost 750 species from 117 genera of Carabidae are known to occur in the Ukraine. Approximately 450 species of ground beetles are registered in the Carpathian region. No less than 300 species of ground beetles are found in the forest zone. Approximately 400 species of Carabidae present in the forest-steppe zone are relatively similar in species composition to those in the forest territories. Some 450 species of Carabidae are inhabitants of the steppe zone. Representatives of many other regions of heterogeneous biotopes such as forest, semi desert, intrazonal, etc. can be found in the steppe areas. The fauna of Carabidae (ca. 100 species) of the lowlands of southern Ukraine (sandy biotopes), situated mostly in the Kherson region, is very peculiar. The fauna of the Crimean mountains contains about 300 species. Conservation measures for the Carabidae are discussed.

## Introduction

The first published observations of ground beetles in the Ukraine appeared at the end of 18th / beginning of the 19th century (Pallas 1776; Steven 1806; Zawadski 1825; Fischer-Waldhaim 1820–1822). Since then the number of publications steadily increased, especially from the second half of the 19th century (Motschulsky 1845, 1850; Chaudoir 1850, 1863; Nowicki 1865, 1873; Hochguth 1871, Łomnicki 1884, 1913; Pliginsky 1911 and others). All these data have been compiled in the fundamental monograph by GG Jacobson (1905–1916). Many further studies on the diversity, ecology and practical importance of Carabidae of the Ukraine had been conducted starting in the early 20th century (Roubal 1924, 1930; Znojko 1927; Lutschnik 1934; Averin 1938; Medvedev 1950, 1954; Arnoldi LV 1953; Arnoldi KV 1958; Kryshtal 1956; Ponomarchuk 1956, 1958, 1963, 1969; Medvedev and Shapiro 1957; Petrusenko AA and Petrusenko SV 1970, 1971, 1973, 1975; Kulyanda 1978; Rizun 1986, 1990, 1994, 1998, 1999, 2003; Eidelberg et al. 1988; Petrusenko et al. 1999; Putchkov 1998, 2008 and many others).

At present, there are more than 1000 literature sources that concern the general biology, systematic and ecology of ground beetle species, recorded from the territory of present-day Ukraine. The checklist of Carabidae of Russia and adjacent territories (Kryzhanovskij et al. 1995) is the largest summary on the species diversity of ground beetles in the Ukraine. In this book, data on the East Carpathian, Crimea and other regions of the Ukraine are presented. Furthermore, a more recent survey in the first volume of the Catalogue of Palearctic Coleoptera (2003) lists nearly 720 species of Carabidae that are indicated for the whole territory of the Ukraine. However, in spite of the fact that these publications span different geographical zones of the Ukraine, the distribution of ground beetles within the country remains poorly studied. Besides, there are nearly thirty species of Carabidae registered in the Ukraine that are not included in the Catalogue of Palearctic Coleoptera, 2003 (marked in this article by*).

The aim of the present paper is to summarise all available data from literature sources and collections and to provide an overview of the present-day species composition and distribution of ground beetles in the Ukraine.

## Material and methods

**Figure 1.**
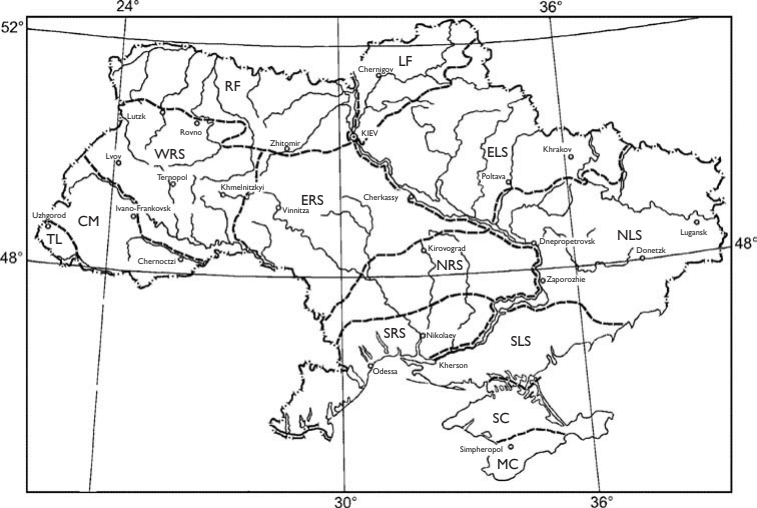
A map of certain geographic regions of the Ukraine: **TL** – Transcarpathian lowland (H < 200 m.); **CM** – Carpathian mountains (H>200 m); **RF**– Right-Dnieper-bank (westwards) of forest zone; **LF** – Left- Dnieper-bank (eastwards) of forest zone; **WRS** – Western part of right- Dnieper-bank (west-westwards) of forest-steppe zone; **ERS** – Eastern part of right- Dnieper-bank (west-eastwards) of forest-steppe zone; **ELS** – Left- Dnieper-bank (eastwards) of forest-steppe zone; **NRS** – Northern subzone of right- Dnieper-bank (westwards) of steppe; **NLS** – Northern subzone of left- Dnieper-bank (eastwards) of steppe; **SRS** – Southern subzone of right- Dnieper-bank (westwards) of steppe; **SLS** – Southern subzone of left- Dnieper-bank (eastwards) of steppe; **SC** – Steppe of Crimean peninsula; **MC** – Crimean Mountains (with south-eastern coastal beach). A list of carabid species recorded from the Ukraine is provided in Appedix 1.

The complete list of Carabidae of the Ukraine (Appendix 1) was compiled on the basis of a critical literature review and collections in several biological institutions in Kiev, Moscow, St.-Petersburg, Budapest, Vienna and Prague, including my own large collection. The tiger beetles (Coleoptera, Cicindelidae), as a separate family (Putchkov and Cassola 2005) is not included in this article. The classification of Carabidae follows Kryzhanovskij et al. (1995) with some additional revision (Catalogue of Palearctic Coleoptera, 2003). The analysis of the distribution of Carabidae in the Ukraine is given on the basis of the whole territory of the country; however special attention was paid to 13 separate specific regions, districts and provinces (Fig. 1).

## Results and discussion

Ground beetles (Carabidae) are one of the largest beetle families in the territory of the Ukraine. There are nearly 750 species from 117 genera present. Such rich biodiversity is due to the large area of the country on one hand, and the heterogeneity of natural conditions of the separate geographical regions on the other hand.

The ground beetle fauna of TL and CM are most diverse in the Ukraine (ca 330 and 400 species from 75 genera were found here, respectively) (Table 1). Eight endemic taxa are registered in the East Carpathians: *Leistus baenningeri* Roubal, 1926, *Leistus ucrainicus* Lazorko, 1954, *Nebria heegeri* Dejean, 1826, *Duvalius transcarpathicus* Shilenkov et Rizun, 1989, *Duvalius ruthenus ruthenus* Reitter, 1878, *Duvalius corpulentus* Weise, 1825, *Trechus pseudomontanellus* Rizun, 1994, *Carabus zawadskyi serriatissimus* Reiter, 1896, *Carabus fabricii ucrainicus* Lazorko, 1951. More than 20 taxa are subendemic for this region (mostly from the genera *Carabus*, *Nebria*, *Trechus* and *Pterostichus*). In addition, more than80 species that are known from the East Carpathians are absent from other geographic regions of the Ukraine. For approximately 50 taxa the Carpathians appear to be the eastern border of their ranges. These are some species belonging to the genera *Nebria*, *Carabus*, *Pterostichus*, *Tachyura*, *Trechus*, andseparate species of *Bembidion*. Most of these species inhabit subalpine and alpine biotopes. Some typical Middle-European species occur in the different types of mountain forests, where they comprise one of the major components of carabid diversity in the Carpathians. At the same time, the fauna of ground beetles in the Carpathians includes also many widespread species that inhabit other forest and forest-steppe areas of the Ukraine.

**Table 1.** The approximate number of genera and species of Carabidae in certain geographic regions of the Ukraine. **TL** Transcarpathian lowland; **CM** Carpathian mountains; **RF** Right-Dnieper-bank; **LF** Left- Dnieper-bank;  **WRS** Western part of  right- Dnieper-bank; **ERS** Eastern part of  right- Dnieper-bank; **ELS** Left- Dnieper-bank;  **NRS** Northern subzone of right- Dnieper-bank; **NLS** Northern subzone of left- Dnieper-bank; **SRS** Southern subzone of right- Dnieper-bank; **SLS** Southern subzone of left- Dnieper-bank; **SC** Steppe of Crimean peninsula; **MC** Crimean Moun-tains; **T** Total.

*Tribes*	*Certain geographic regions of the Ukraine (number genera/species)*															
	*TL*	*CM*	*Forest zone*			*Forest-steppe zone*				*Steppe zone*						*MC*
			*T*	*RF*	*LF*	*T*	*WRS*	*ERS*	*ELS*	*T*	*NRS*	*NLS*	*SRS*	*SLS*	*SC*	
1. Omophronini	*1/1*	*1/1*	*1/1*	1/1	1/1	*1/1*	1/1	1/1	1/1	*1/1*	1/1	1/1	1/1	1/1	1/1	*1/1*
2. Nebriini	*2/6*	*2/16*	*2/6*	2/6	2/6	*2/8*	2/8	2/5	2/5	*2/4*	2/4	2/2	1/3	1/1	1/1	*2/4*
3. Notiophilini	*1/5*	*1/7*	*1/6*	1/6	1/4	*1/7*	1/6	1/6	1/7	*1/6*	1/5	1/6	1/6	1/4	1/2	*1/5*
4. Carabini	*2/22*	*2/30*	*2/20*	2/14	2/18	*2/32*	2/30	2/21	2/23	*2/26*	2/22	2/19	2/17	2/16	2/11	*2/14*
5. Cychrini	*1/1*	*1/2*	*1/1*	1/1	1/1	*1/2*	1/2	1/2	1/1	*1/1*	1/1	–	–	–	–	*–*
6. Elaphrini	*2/5*	*2/5*	*2/5*	2/5	2/3	*2/6*	2/5	2/4	2/6	*1/2*	1/2	1/1	1/1	1/1	1/2	*1/2*
7. Loricerini	*–*	*1/1*	*1/1*	1/1	1/1	*1/1*	1/1	1/1	1/1	*–*	–	–	–	–	–	*–*
8. Scaritini	*–*	*–*	*–*	–	–	*–*	–	–	–	*1/2*	1/1	1/1	1/2	1/2	1/2	*1/1*
9. Clivinini	*1/2*	*1/2*	*1/2*	1/2	1/2	*1/2*	1/2	1/2	1/2	*1/3*	1/2	1/2	1/3	1/3	1/3	*1/2*
10. Dyschiriini	*1/9*	*1/13*	*1/10*	1/10	1/10	*1/17*	1/16	1/12	1/15	*1/22*	1/14	1/16	1/25	1/20	1/22	*1/6*
11. Broscini	*1/1*	*1/1*	*1/2*	1/2	1/1	*1/1*	1/1	1/1	1/1	*1/2*	1/1	1/2	1/2	1/2	1/2	*1/2*
12. Apotomini	*–*	*–*	*–*	–	–	*–*	–	–	1/1	*1/1*	–	1/1	1/1	1/1	1/1	*–*
13. Trechini	*5/5*	*8/23*	*6/7*	6/7	6/6	*6/9*	6/9	6/6	4/4	*3/3*	3/3	3/3	2/2	2/2	2/2	*6/8*
14. Tachyiini	*2/3*	*2/3*	*2/2*	2/2	2/2	*2/4*	2/4	2/2	2/2	*2/5*	2/2	1/2	2/4	2/5	2/6	*2/3*
15. Bembidiini	*2/52*	*2/70*	*2/40*	2/38	2/31	*2/59*	2/57	2/36	2/33	*2/34*	2/28	2/28	2/30	2/31	35	*2/33*
16. Pogonini	*–*	*–*	*–*	–	–	*–*	–	1/1	1/2	*3/14*	1/2	1/8	3/11	3/14	3/13	*–*
17. Patrobini	*1/2*	*1/3*	*1/1*	1/1	1/1	*1/1*	1/1	1/1	1/1	*–*	–	–	–	–	–	*–*
18. Deltomerini	*–*	*1/1*	*–*	–	–	*–*	–	–	–	*–*	–	–	–	–	–	*–*
19. Pterostichini	*6/25*	*6/35*	*5/26*	5/24	5/23	*6/31*	6/30	5/22	4/21	*4/22*	4/18	4/21	3/19	3/19	3/18	*3/14*
20. Sphodrini	*2/5*	*3/8*	*3/7*	3/7	2/7	*3/8*	3/8	2/6	2/6	*3/11*	3/5	3/5	3/8	3/10	3/10	*3/14*
21. Platinini	*8/24*	*9/28*	*8/30*	8/28	7/26	*8/28*	8/28	7/21	7/21	*5/15*	5/14	5/12	5/15	4/13	3/12	*6/15*
22. Zabrini	*3/31*	*3/35*	*3/33*	3/33	3/33	*3/38*	3/36	3/31	3/33	*3/36*	3/28	3/25	3/28	3/33	3/35	*3/30*
23. Harpalini	*10/57*	*10/55*	*10/50*	9/49	8/46	*10/64*	10/64	8/50	8/68	*17/ca 130*	11/72	10/75	14/ca 100	14/ca 110	16/ca 120	*11/ ca70*
24. Perigonini	*–*	*–*	*–*	–	–	*–*	–	–	–	*–*	–	–	–	–	1/1	*–*
25 Panageini	*1/2*	*1/2*	*1/2*	1/2	1/2	*1/2*	1/2	1/2	1/2	*1/2*	1/2	1/2	1/2	1/2	1/1	*1/1*
26 Callistini	*3/9*	*2/8*	*2/7*	2/7	2/6	*3/9*	2/9	3/9	3/9	*4/15*	3/10	3/10	3/14	4/15	4/14	*3/11*
27 Oodini	*1/2*	*1/2*	*1/2*	1/2	1/2	*1/2*	1/2	1/2	1/2	*1/2*	1/2	1/2	1/2	1/2	1/2	*1/2*
28 Licinini	*2/6*	*2/6*	*2/10*	2/7	2/10	*2/10*	2/9	2/8	2/10	*2/9*	2/9	2/9	2/9	2/8	2/7	*2/7*
29 Masoreini	*–*	*–*	*1/1*	1/1	1/1	*1/1*	1/1	1/1	–	*1/1*	–	–	–	1/1	–	*1/1*
30.Corsyrini	*–*	*–*	*–*	–	–	*–*	–	–	–	*1/1*	–	1/1	1/1	–		
31 Odacanthini	*1/1*	*1/1*	*1/1*	1/1	1/1	*1/1*	1/1	1/1	1/1	*1/1*	1/1	1/1	1/1	1/1	1/1	*1/1*
32 Lebiini	*9/22*	*9/20*	*8/19*	8/19	8/18	*8/20*	9/25	8/15	8/20	*8/ca 40*	8/18	8/17	8/26	8/30	8/35	*9/25*
33 Dryptini	*1/1*	*1/1*	*1/1*	1/1	1/1	*1/1*	1/1	1/1	1/1	*2/2*	1/1	1/1	2/2	2/2	2/2	*2/2*
34 Zuphiini	*1/1*	*–*	*1/1*	–	1/1	*–*	–	–	1/1	*2/3*	1/1	1/2	2/3	2/3	2/2	*2/3*
35 Brachinini	*1/1*	*1/3*	*1/2*	1/2	1/1	*2/4*	2/3	2/4	1/3	*2/16*	2/4	2/5	2/10	2/11	2/15	*2/11*
*Totals*	*71/327*	*75/390*	*70/300*	70/280	69/265	*75/390*	73/365	69/275	65/310	*76/ca 450*	64/273	63/275	68/ca 340	70/ca 360	70/ca 370	*68/ca 280*

The fauna of forest areas of the Ukraine is represented by no less than 300 species from 70 genera and is characterized by considerable similarity in species composition in all separate regions and zones (Table 1). It appears also similar to those from other northern areas of eastern Europe. Endemics are absent from forest area of the Ukraine, although about 10 species are present only in forest areas of the country. There are some boreal taxa of carabids that are registered only in the north-western part of the Ukraine (some species of *Carabus*, Miscodera*, *Agonum*, *Trechus*, *Pterostichus*). The territory of RF is slightly richest in terms of species diversity of Carabidae then LF of the forest zone (Table 1).

Ground beetles of the present-day forest-steppe zone are represented by nearly 400 species from 75 genera (Table 1). The fauna of this zone is not typically transitional from forest to steppe. Obviously, the species composition of ground beetles in the forest-steppe can be characterized as quite distinct, although with some similarities to the forest zone. The variety and number of typical forest species (especially hygrophilous and mesophilous ones) exceeds the number of steppe-specific inhabitants. The territory of WRS is the richest (365 species from 73 genera) in terms of species diversity of Carabidae as compared to that of ERS (310 species from 65 genera) (Table 1). Forest species are more common in the western region between the Dniester and South Bug rivers. Four species - *Carabus sibiricus rybinskii* Reitter, 1896, *Laemostenus tichyi* Kult, 1946 (both are endemics of the Ukraine), *Poecilus szepligetii* Csiki, 1908and *Aptinus bombarda* Illiger, 1800occur only in the western area. Moreover, some ground beetles (*Carabus excellens* Fabricius, 1798, *Carabus marginalis* Fabricius, 1794, *Carabus scabriusculus* Olivier, 1795, some *Calathus*)aremore abundant in the forest-steppe zone than in northern or southern regions. However there are many forest species of Carabidae that occur in WRS that are absent from the ERS. The composition Carabidae in the ELS is characterized by an increasing number of mesoxerophilous species, which are more common in the steppes than in the forest-steppe(*Harpalus*, *Cymindis*) (Table 1).

The ground beetle fauna of the steppe area of the Ukraine is the richest in species diversity and is characterized by the presence of approximately 450 species from 76 genera (Table 1). The taxonomic structure of ground beetles of the steppe is very diverse due to the heterogenous origin of steppe Carabidae fauna. The occurrence of many extrazonal (forest or semi-desert) and intrazonal (littoral, halophilous) species in the steppe region zone makes it difficult to characterize the general composition of the carabid fauna. As a whole, about 100 taxa of ground beetles in the fauna of the Ukraine occur exclusively in the steppe area (especially the genera *Scarites*, *Apotomus*, *Zuphium*, many Harpalini, Zabrini and some species of *Poecilus*, *Chlaenius* and *Brachinus*). However, the majority of ground beetles in this area is formed by typical steppe or Mediterranean taxa (mostly from the tribes Harpalini, Zabrini, Lebiini). Some forest and forest-steppe species (tribes Nebriini, Carabini, Platinini) are more common in the northern subzone (NRS and NLS) of the steppe zone. In addition, anthropogenic factors have supported predominance of some widespread mesophilous species in this subzone.

The ground beetle fauna of the steppe area of NLS is similar as a whole to that of the NRS; however it is characterized at the same time by the predominance of typical steppe taxa. Only the district of the Donetskyi heights (ridge) is characterized by more mesophilous elements including polytopic and forest species (some *Carabus*, *Pterostichus* and *Agonum*). At the same time, the occurrence of typical steppe taxa, including separate inhabitants of Caucasian and Kazakhstanian steppes (*Poecilus anodon* Chaudoir, 1868, *Poecilus lyroderes* Chaudoir, 1846, *Curtonotus propinquus* Menetries, 1832, and some *Cymindis* species) could be observed in this region. Possibly, earlier, the Donetskyi ridge was characterised by a ground beetle fauna transitional between forest-steppe and steppe zones. Present-day diversity of Carabidae of this region is relatively closer to that of typical steppe fauna.

The southern steppe subzone (SRS and SLS) is characterized by the prevalence of xerophilous and mesoxerophilous species from the tribes Harpalini and Lebiini, while relatively mesophilous taxa occur more exceptionally in river valleys, ravines or in agricultural biotopes. The occurrence of some mesohygrophilous species in the steppe is usually related to irrigation.

The majority of littoral and halophilous species (tribes Clivinini, Bembidiini, Tachyini, Pogonini and Stenolophina) occur in river valleys, coastal beaches of gulfs, lakes, estuaries and other water basins. The ground beetle fauna of seashores and estuaries is characterized by the prevailence of many species that are absent from other regions of the Ukraine (some *Dyschirius*, *Tachys*, *Bembidion*, *Acupalpus*, *Trichocellus* etc.).

Quite specific, although poor in species number (no more than 100), is the carabid fauna of sandy habitats in the lowlands of Dnieper River (Kherson region, Oleshie). It is represented by both typical steppe species and psammophilous and some semi-desert elements (*Cymindis medvedevi* Kryzhanovskij et Emetz, 1973, *Corsyra fusula* Fischer von Waldheim, 1820*, *Polystichus connexus* Fourcroy, 1785*, *Parazuphium chevrolatii* Castelnau, 1833*). Many halophilous and littoral species from the tribes Pogonini, Scaritini, Bembidiini and Harpalini also occur here. In addition, some typical forest inhabitants were also recorded from this region (*Carabus*, *Pterostichus*, *Agonum* occurring in groves).

The ground beetle fauna of the Crimean Peninsula is one of the most specific in the Ukraine (about 390 species from 74 genera). There are some typical inhabitants of steppe and halophitic biotopes of the plains of Crimea (near 370 species): *Calosoma*, *Carabus*, *Poecilus*, *Amara*, many Harpalini and Cymindina (Table 1). On the Kerch Peninsula some relatively forest mesophilous species occur: *Carabus cancellatus* Illiger, 1798, *Leistus ferrugineus* Linnaeus, 1758, *Pterostichus niger* Schaller, 1783 and *Pterostichus anthracinus* Illiger, 1798. This confirms the presence of arboreal areas in the ancient past.

The fauna of MC (no less than 280 species from 68 genera) has quite a different composition from that of the plain regions of the Crimea (Table 1). It is characterized by some Crimean endemics (about 15 taxa, e.g. some cave species from the genera *Pseudophaenops* and *Taurocimmerites*, as well as *Carabus gyllenhali* Fischer von Waldheim, 1827, *Carabus hungaricus gastridulus* Fischer von Waldheim, 1823, *Carabus perrini* *planus* Gehin, 1885, *Carabus sabrosus tauricus* Bonelli, 1811, *Trechus lioplerus jailensis* Winkler, 1911, *Bembidion iphigenia* Netolitzky, 1931, *Laemostenus jailensis* Breit, 1911, *Cymindis vagemaculata* Breit, 1914). Some taxa are subendemic to MC and are recorded from the Caucasus as well (*Leistus caucasicus* Chaudoir, 1876, *Carabus sibiricus bosphoranus* Fischer von Waldheim, 1823, *Bembidion lederi* Reitter, 1888, *Laemostenis sericeus tauricus* Dejean, 1828) or in other southern European countries (*Laemostenus cimmerius* Fischer von Waldheim, 1823*, *Laemostenus venustus* Clairville, 1828*, *Cymindis ornata* Fischer von Waldheim, 1824, *Cymindis scapularis* Schaum, 1857*). However, the bulk of the ground beetle fauna of the Crimea Mountain consists of taxa that are widespread in the Mediterranean region and/or in forest-steppe areas of the Ukraine.

Special attention should be paid to the fauna of anthropogenic landscapes of the Ukraine. In agricultural habitats, the species composition of ground beetles is relatively uniform throughout the different geographical regions. Agrocenoses are generally poor in species richness consisting of some 70–100 widely distributed common species, but the abundance of some of these is much higher than in natural biotopes. The core faunal composition consists of approximately 20 widespread (mainly polytopic) species from the genera *Amara*, *Bembidion*, *Harpalus*, *Poecilus* and *Pterostichus*. The fauna of urban territories (for example cities) is rather impoverished as a rule and consists of some 10–15 polytopic species.

Currently ten species of ground beetles (*Calosoma sycophanta* Linnaeus, 1758 *Carabus bessarabicus* Fischer von Waldheim, 1823, *Carabus estreicheri* Fischer von Waldheim, 1822, *Carabus hungaricus* Fabricius, 1792, *Carabus scabrosus tauricus* Bonelli, 1811, *Carabus stscheglowi* Mannerheim, 1827, *Pseudophaenops jacobsoni* Pliginsky, 1913, *Taurocimmerites dublanskii* Belousov, 1998, *Carterus dama* Rossi, 1792 and *Parazuphium chevrolatii* Castelnau, 1833*) are protected and enlisted in the “Red Book of Ukraine, 2009”. Most of these species are rare or vulnerable in the Ukraine; moreover the last two are cave endemics of the Crimea. Additionally, three species that occur in the Ukraine (*Carabus hampei* Kuster., 1846, *Carabus zawadskyi* Kraatz, 1854, *Carabus variolosus* Fabricius, 1794) are included in the European Data Red List as vulnerable. In general, approximately 40 species of Carabidae in total need to be protected in the Ukraine (Table 2).

**Table 2.** Rare and little-known species of Carabidae of the Ukrainian fauna.

*N*	*Species*	*Region, biotope*	*N*	*Species*	*Region, biotope*
1	*Leistus caucasicus* Chaudoir, 1876	Crimea Mnts, beech forest	20	*Poecilus anodon* Chaudoir, 1868	south-east, steppe
2	*Leistus baenningeri* Roubal,1926	Carpathian, subalpine zone	21	*Laemostenus jailensis* Breit, 1914	Crimea Mnts, subalpine zone
3	*Nebria heegeri* Dejean, 1826	Carpathian, subalpine zone	22	*Taphoxenus gigas* Fischer von Waldheim, 1823	south steppe
4	*Carabus menetriesi* Faldermann, 1827	forest zone, swampy	23	*Bradycellus caucasicus* Chaudoir, 1846	forest zone
5	*Carabus intricatus* Linnaeus, 1761	west Ukraine, forest	24	*Parophonus planicollis* Dejean, 1829*	south steppe
6	*Carabus ullrichi* Germar, 1824	west -south part, Carpathian, forest zone	25	*Carterus angustipennislutschniki* Zamotailov, 1988	East Crimea, steppe
7	*Carabus nitens* Linnaeus, 1758	north of Ukraine, forest	26	*Ditomus calydonius oriens* Rossi, 1790	south steppe
8	*Carabus fabricii ucrainicus* Lazorko, 1951	Carpathian, alpine zone	27	*Eucarterus sparsutus* Reitter, 1898	south steppe
9	*Elaphrus uliginosus* Fabricius, 1792*	Forest and east of forest steppe zones, Crimea	28	*Epomis circumscriptus* Duftschmidt, 1812	south-east, littoral
10	*Scarites laevigatus* Fabricius, 1792	south steppe	29	*Chlaenius alutaceus* Gebler,1829	forest zone, swampy
11	*Apotomus testaceus* Dejean, 1825	south steppe	30	*Chlaenius costatulus* Motschulsky, 1859*	forest zone, swampy
12	*Duvalius transcarpathicus* Shilenkov et Rizun, 1989	Carpathian, subalpine zone	31	*Masoreus wetterhali* Gyllenhal, 1813	forest and forest-steppe zones
13	*Pseudaphaenops tauricus* Winkler, 1912	caves of CrimeaMnts	32	*Cymindis vagemaculata* Breit, 1914	Crimea mnts, beech’s forest
14	*Trechus fontinalis* Rybinsky, 1900	Carpathian, subalpine zone	33	*Cymindis medvedevi* Kryzhanovskij et Emetz, 1973	sand of south steppe (Kherson reg.)
15	*Trechus plicatulus* Miller, 1868	Carpathian, subalpine zone	34	*Zuphium olens* Rossi, 1790*	south steppe
16	*Bembidion lederi* Reitter, 1888	Crimea Mnts, near streams	35	*Brachinus bipustulatus* Quensel, 1806	south steppe, Crimea
17	*Brachinus iphigenia* Netolitzky, 1931	Crimea Mnts, near streams	36	*Brachinus hamatus* Fischer von Waldheim, 1828*	south steppe, Crimea
18	*Pogonus cumanus* Lutschnik, 1916	south-east, halobiont	37	*Mastax thermarum* Steven, 1806*	south-west, forest zone
19	*Pedius longicollis* Duftschmidt, 1812	south-east, steppe	38	*Aptinus bombarda* Illiger, 1800	south-west region

## Conclusions

I conclude that the overall species composition of the ground beetle fauna of the Ukraine is well studied. Therefore, finding new taxa in any part of the Ukraine is not likely to happen in the near future. Regions where new taxa for the Ukraine could be found are mainly boundary regions of the country (the Carpathians, Crimea, north and east regions), as well as in errors of some findings in the past (for example, mis-identifications of some taxa or species with wrong labels).

Poorly answered questions that remain include the origins of carabid fauna of the Ukraine. Moreover it is necessary to study the preimaginal stages of ground beetles. For example the larvae of only 360 species from 86 genera are described, representing only 45% of carabid species from the Ukraine. Further studies on the bionomics of single species require urgent attention. This applies in particular to ecologically related, non-competitive species occurring in common biotopes, e.g. many littoral species of *Bembidion*, *Dyschirius*, *Acupalpus* or some steppe species of the Harpalini tribe.

## Appendix 1

Checklist of ground beetle species recorded from the Ukraine. (doi: 10.3897/zookeys.100.1545.app) File format: Microsoft Word (doc).

**Explanation note:** The additional file contains a list of all ground beetle species recorded from the Ukraine.

Copyright notice: This dataset is made available under the Open Database License (http://opendatacommons.org/licenses/odbl/1.0/). The Open Database License (ODbL) is a license agreement intended to allow users to freely share, modify, and use this Dataset while maintaining this same freedom for others, provided that the original source and author(s) are credited.
